# Long-term excess mortality after chronic subdural hematoma

**DOI:** 10.1007/s00701-020-04278-w

**Published:** 2020-03-07

**Authors:** Minna Rauhala, Pauli Helén, Karri Seppä, Heini Huhtala, Grant L. Iverson, Tero Niskakangas, Juha Öhman, Teemu M. Luoto

**Affiliations:** 1 Department of Neurosurgery, Tampere University Hospital and Tampere University, Tampere, Finland; 2 Finnish Cancer Registry, Institute for Statistical and Epidemiological Cancer Research, Helsinki, Finland; 3 Faculty of Social Sciences, Biostatistics Group, Tampere University, Tampere, Finland; 4 Department of Physical Medicine and Rehabilitation, Harvard Medical School, Boston, MA USA; 5 Spaulding Rehabilitation Hospital and Spaulding Research Institute, Boston, MA USA; 6 Home Base, A Red Sox Foundation and Massachusetts General Hospital Program, Boston, MA USA; 7 Faculty of Medicine and Life Sciences, Tampere University, Tampere, Finland

**Keywords:** Subdural hematoma, chronic, Mortality, excess, Causes of death, Survival, Mortality, excess

## Abstract

**Objective:**

To assess possible long-term excess mortality and causes of death of patients with chronic subdural hematoma (CSDH).

**Methods:**

A retrospective study (1990–2015) of adult patients (*n* = 1133, median age = 76 years old, men = 65%) with CSDH identified by ICD-codes and verified by medical records. All patients were followed until death or the end of 2017. Cumulative relative survival ratios and relative excess risks of death (RER) were estimated by comparing patients’ mortality with that in the entire regional matched population. The causes of death were compared with a separate reference group formed by randomly choosing sex, age, and calendar time matched controls (4 controls per each CSDH patient).

**Results:**

The median follow-up time was 4.8 years (range = 0–27 years), and 710 (63%) of the patients died (median age at death = 84 years old). The cumulative excess mortality was 1 year = 9%, 5 years = 18%, 10 years = 27%, 15 years = 37%, and 20 years = 48%. A subgroup of CSDH patients (*n* = 206) with no comorbidity had no excess mortality. Excess mortality was related to poor modified Rankin score at admission (RER = 4.93) and at discharge (RER = 8.31), alcohol abuse (RER = 4.47), warfarin (RER = 2.94), age ≥ 80 years old (RER = 1.83), non-operative treatment (RER = 1.56), and non-traumatic etiology (RER = 1.69). Hematoma characteristics or recurrence were unrelated to excess mortality. Dementia was the most common cause of death among the CSDH patients (21%) and the third most common cause in the reference group (15%, *p* < 0.001).

**Conclusions:**

Patients with CSDH have continuous excess mortality up to 20 years after diagnosis. Patient-related characteristics have a strong association with excess mortality, whereas specific CSDH-related findings do not. CSDH patients have an increased risk for dementia-related mortality.

## Introduction

Chronic subdural hematoma (CSDH) is a common disease in neurosurgical practice among elderly patients [1, 3, 27]. The reported annual incidence of CSDH has ranged widely across studies, from 1.7 to 20.6 per 100,000—and the overall incidence is increasing as the global population becomes progressively older [38]. In a prior study using this same patient cohort, we reported that during a 26-year period between 1990 and 2015, the overall incidence doubled from 8.2 to 17.6/100,000/year in the Pirkanmaa region, Finland [32]. The incidence per 100,000 person-years remained quite stable among adults younger than 70 years, whereas the incidence nearly tripled among the population 80 years or older. The global population of people aged 80 and older is expected to more than triple between 2015 and 2050 [17]. Consequently, CSDH is a condition of growing importance.

CSDH has been considered to be relatively benign, but during the last years, it has been recognized to have worse outcome than earlier assumed [11, 25, 29]. It has been speculated that CSDH may be a sentinel health event, and a harbinger of subsequent morbidity and mortality [3, 11, 29]. Age-related brain degeneration with an enlarging potential subdural space is assumed to be an important risk factor for CSDH [22, 24, 37]. Conversely, CSDH itself has been associated with a significant increase in the degree of brain atrophy post-CSDH [4]. Other well-known risk factors for CSDH are trauma [26], alcohol overuse [28], and antithrombotic therapy [6, 15, 23, 30].

Reported mortality rates after CSDH vary widely across studies, and a 1-year mortality rate of up to 32% has been reported [29]. In general, CSDH patients are from an age group with high baseline expected mortality. It is not possible to draw reliable conclusions on the excess mortality related to CSDH without comparing these patients to a matched sample from the general population. To date, only four studies (Table 1) have compared mortality after CSDH with anticipated survival [11, 16, 25, 29]. All of these studies have shown varied excess mortality, but the follow-up periods have extended only up to 8 years. Furthermore, there are no prior studies reporting long-term mortality in CSDH patients compared with a matched sample from the general population in an unselected, population-based series. Additionally, only two studies have previously reported the causes of death after a diagnosis of CSDH [19, 25].

**Table 1:** Summary of studies on long-term excess mortality in chronic subdural hematoma patients

Authors	Country	Time period	*n*	Age mean, y (range)	Follow-up period median, y (range for survivors)	Mortality (%)		Control data	Excess mortality
						6 months	1 year		
Miranda et al. 2011^7^	USA	2000–2008	209	80.6 (65–96)	1.45 (N/A-8.3)	26.3	32	Center of Disease Control and Prevention data	• Excess mortality up to 1 year beyond diagnosis • Median survival: o CSDH 4.4 year o Anticipated actuarial survival 6 year
Dumont et al. 2013^8^	USA	1996–2010	287	75 (55-N/A)	2.3 (0.5–14)	N/A	30	Center of Disease Control and Prevention data	• 1 year standardized mortality ratio: o 55–64 years:17 o 65–74 years:8.1 o 75–84 years:3.4 o ≥ 85 years:2.9 • Median survival: 4.0 ± 0.5y
Manickam et al. 2016^9^	Australia	2006–2011	155	69.3 (18-N/A)	5.2 (N/A-14.19)	14.19	20.35	Australian Bureau of Statistics, and the Registry of Births, Deaths and Marriages	• Excess mortality throughout follow-up. • Average long-term survival: o CSDH: 5.29 ± 0.59 year o Actuarial data: 17.74 ± 1.8 year
Guilfoyle et al. 2017^19^	UK	2004–2007	215	78 (35–95)	N/A (8–10)	Drain group: 8.6 No drain group: 18.1	N/A	Cohorts of the general population with the same number of cases and identical age and sex profiles as the drain and no drain groups (Human Mortality Database)	• 5 year cumulative excess mortality: o Drain group: 10.2% o No drain group: 22.4%
Present study	Finland	1990–2015	1133	73 (22–99)	4.8 (2–27)	9.5	13.7	The population of Pirkanmaa region stratified by sex, age, and calendar year (Statistics Finland)	• Cumulative excess mortality: o 1 year: 9% o 5 years: 18% o 10 years: 27% o 15 years: 37% o 20 years: 48%

CSDH = chronic subdural hematoma; N/A = not available

The objective of this study was to examine the possible long-term excess mortality related to CSDH, and the causes of death after a diagnosed CSDH. A large unselected, population-based CSDH patient cohort was compared with the general population from the same region, matched by sex, age, and calendar time. The causes of death of the CSDH patients were compared with a separate matched reference group.

## Methods

### Material and ethical aspects

The study was conducted in the Department of Neurosurgery at the Tampere University Hospital (Tampere, Finland). All adult patients (≥ 18 years old Pirkanmaa residents) with a diagnosis of CSDH between 1990 and 2015 were retrospectively identified using the hospital’s patient administrative databases. The cases were identified using International Classification of Diseases (ICD) codes for traumatic and non-traumatic subdural hematomas (SDHs). Verified cases were classified by SDH type (acute, subacute, chronic, and hygroma) by reviewing all the medical records. Exclusion criteria were acute or subacute SDH (< 3 weeks after head trauma), hygroma (a collection of subdural cerebrospinal fluid without any signs of blood), and any form of intracranial surgery within 12 months preceding the CSDH diagnosis.

The dates and causes of deaths were obtained from Statistics Finland (Helsinki, Finland). The Finnish official cause of death statistics are, in practice, 100% complete in relation to the cause and date of death. The entire Pirkanmaa population matched by sex, age, and calendar time was used for the excess mortality analysis. For the cause of death comparison, a separate reference group was formed by randomly choosing 4:1 sex, age (± 6 months), and calendar time–matched control subjects from Pirkanmaa for each CSDH patient. The reference group (*n* = 4532) was obtained from the Statistics Finland.

The Pirkanmaa region is a geographically well-defined area with both rural and urban areas that holds one of Finland’s five neurosurgical departments (Department of Neurosurgery, Tampere University Hospital, Tampere, Finland). All neurosurgical cases of the Pirkanmaa region are referred to the Tampere University Hospital. Over 9% of the Finnish population lives in the Pirkanmaa region. The population increased from 427,223 in 1990 to 506,114 in 2015. The population over 80 years old has almost doubled from 13,565 to 26,417 during the study period.

### Data collection

A detailed and structured data collection was performed from medical records. CT scans or MR images were not separately inspected. Patients were stratified into three groups according to age:(1) 18–59 years, (2) 60–79 years, and (3) ≥ 80 years. The age categories were formed a priori on the basis of previous literature, convenience and the ease of presenting the results. The data collection included the following: comorbidities, medication, possible trauma, symptoms, neurological condition assessments based on both the Glasgow Coma Scale (GCS) and the modified Rankin Scale (mRS) score at admission, and for the operative group also at discharge. CSDH-related findings collected were localization (unilateral/bilateral) and hematoma thickness divided into three groups (≤ 15 mm, 16–25 mm, and > 25 mm) selected before the data collection was started. Operation details were collected. CSDH recurrence was defined as an ipsilateral hematoma needing re-operation within 2 years of the original operation. All patients were followed until death or the end of year 2017.

### Survival analysis

The variables chosen for survival analysis were sex, age groups, and variables known to be CSDH risk factors (trauma, chronic alcohol abuse, and antithrombotic medication). The effect of neurological condition, treatment group (operative versus non-operative), and hematoma recurrence were analyzed.

### Statistical analyses

SPSS (IBM SPSS Statistics for Windows, Version 25.0, Armonk, NY, USA) was used for data analyses. Survival analyses were conducted using the statistical software R (version 3.6.0) with popEpi package (version 0.4.7). Descriptive statistics [frequency (*n*), percentage, median, interquartile range, range] were used to describe variable and subgroup characteristics. The Chi square test was used to compare differences between groups. The statistical significance level was set at *p* < 0.05.

The cumulative relative survival ratio (CRSR) summarizes patients’ excess risk of death due to the disease by comparing the survival of patients to that of the matched general population (the population of Pirkanmaa region stratified by sex, age, and calendar year). CRSRs were estimated by using the Ederer II method [13, 34]. To compare differences in relative survival adjusted for age, sex, and follow-up time, we estimated relative excess risk (RER) of death by using Poisson regression [10]. Each model included sex, age at diagnosis (4 groups: 0–59, 60–69, 70–79, and 80+ years), and 5 intervals of follow-up time after diagnosis (0 to < 1 year, 1 to < 5 years, and three 5-year intervals from 5 to 20 years) in addition to a risk factor.

### Data availability statement

The data that support the findings of this study are available from the corresponding author upon reasonable request.

## Results

### Characteristics

A total of 1133 patients with CSDH were identified, 736 (65%) were men. The median age for CSDH diagnosis was 76 years, and women were older than men (79 vs. 75 years). Median follow-up time was 4.8 years, with a minimum of 0 days and maximum of 27 years. Median follow-up time for survivors was 6.6 years, with a minimum of 2 years. No patients were lost from follow-up.

Of all the patients, 965 (85%) were operated. The indication for surgery was based on imaging and symptoms attributable to the mass effect of the hematoma. Operatively treated patients were slightly younger than non-operatively treated (median age 76 vs. 79 years, *p* = 0.001). At least one comorbidity was reported by 82% of the patients in both treatment groups. Antithrombotic medication was used by 42% of the patients. Two or more comorbidities were reported by 38% of operative group patients and 48% of non-operative group patients (*p* = 0.015), three or more by 13% and 15% (*p* = 0.052), respectively. Non-operatively treated CSDH patients more often had previously diagnosed dementia (16% vs. 8%, *p* = 0.001). The overall prevalence of dementia in CSDH patients aged 70 years or older was 12%. Significant differences were also noted in admission mRS; operatively treated patients had worse neurological disability [mRS scores of 4–5 were found in 52% of the operative group (*n* = 506/965) versus 28% of the non-operative group (*n* = 47/168), *p* < 0.001]. Operatively treated patients more often had headache or localizing neurological deficits. The characteristics of the entire sample and treatment subgroups are presented in Table 2. We have previously published the details of CSDH patients from this same patient cohort stratified by gender, age groups, and time periods [32].

**Table 2:** Characteristics of all chronic subdural hematoma patients and treatment subgroups

	Total sample *n* = 1133		Non-operative treatment *n* = 168		Operative treatment *n* = 965		*p* value
	*n*	%	*n*	%	*n*	%	
Median age, years, IQR	76	67–83	79	68–86	76	66–82	0.001
Sex							
Men	736	65.0	102	60.7	634	65.7	0.21
Traumatic etiology	672	59.3	107	63.7	565	58.5	0.21
Comorbidity							
Cardiovascular disease	644	56.8	96	57.1	548	56.8	0.93
Diabetes	178	15.7	27	16.1	151	15.6	0.89
Chronic alcohol abuse	126	11.1	20	11.9	106	11.0	0.73
Cerebrovascular disease	124	10.9	24	14.3	100	10.4	0.13
Dementia	99	8.8	26	15.7	73	7.6	0.001
Pulmonary disease	68	6.0	16	9.5	52	5.4	0.05
Epilepsy	32	2.8	3	1.8	29	3.0	0.38
Neurodegenerative disease	20	1.8	7	4.2	13	1.3	0.01
Hydrocephalus	9	0.8	0	0	9	0.9	0.21
Medication							
Antiplatelet	268	23.7	36	21.4	232	24.0	0.46
Warfarin	187	16.5	26	15.5	161	16.7	0.69
Warfarin AND antiplatelet	23	2.0	2	1.2	21	2.2	0.40
Admission GCS							
13–15	1007	88.9	157	93.5	850	88.1	0.04
9–12	91	8.0	8	4.8	83	8.6	0.91
3–8	35	3.1	3	1.8	32	3.3	0.29
Admission mRS 0–3	580	51.2	121	72.0	459	47.6	< 0.001
Symptoms							
Hemiparesis	444	39.2	9	5.4	435	45.1	< 0.001
Vertigo or postural instability	408	36.0	35	20.8	373	38.7	< 0.001
Disorientation/memory impairment	390	34.4	54	32.1	336	34.8	0.50
General malaise	367	32.4	50	29.8	317	32.8	0.43
Headache	340	30.0	28	16.7	312	32.3	< 0.001
Aphasia	245	21.6	9	5.4	236	24.5	< 0.001
Seizure	117	10.3	28	16.7	89	9.2	0.003
Nausea and/or vomiting	60	5.3	7	4.2	53	5.5	0.48

*IQR* Interquartile range; *GCS* Glasgow Coma Scale; *mRS* Modified Rankin Scale

Most operations used local anesthesia (*n* = 828; 86%) via one burr hole, and the hematoma was removed through irrigation. A subdural drain was inserted in 59 patients (6%). The drain was kept below the head level with no suction for 24–48 h. Subgaleal drains were not used. Only one patient underwent craniotomy as the primary surgery. The patients were actively mobilized directly after the operation. Recurrent hematoma was treated surgically for 273 cases (28%, median age 76 years). The reason for non-operative treatment for majority of the cases was that the CSDH did not cause neurological signs or significant symptoms. Only 7 patients were not offered surgery because they presented in a moribund state, and the analyses have been done also by excluding these patients. Most non-operatively treated patients were not admitted to a neurosurgical clinic. Non-operative treatment included discontinuation of possible antithrombotic medication, active mobilization, and follow-up CT-scans (routinely or for emerging new symptoms).

### Mortality after diagnosis of CSDH

By the end of the follow-up period, 710 (63%) of the 1133 patients had died, 449 of men (61%), and 261 of women (66%). Median age at death was 84 years (IQR 76–89 years), 83 years for men, and 86 years for women. Similarly, the median age at death was 84 years in the operative group and 85 years in the non-operative group. The 30-day and 6-month mortalities after diagnosis of CSDH were 3% and 10%, respectively. The overall 1-year and 2-year mortality rates were 14% and 22%, respectively. There was no significant difference in mortality between men and women. One-year mortality was 12% in the operative group (*n* = 965) and 21% in the non-operative group (*n* = 168; *p* = 0.003). After the patients (*n* = 7) who were not offered surgery, because they presented in a moribund state, were withdrawn from the non-operative group, the non-operative 1-year mortality was 18% (operative vs. non-operative: *p* = 0.053). One-year mortality was 5% among the patients under the age of 60, and 22% among those aged 80 and older (*p* < 0.001). A subgroup of patients with no comorbidities had a 1-year mortality of only 3% (*n* = 206, median age 72 years, IQR 61–78 years). Mortality rates are presented in Table 3. For comparison, the reference group mortality rates also are reported in Table 3.

**Table 3:** Mortality after diagnosis of CSDH stratified by sex, age, treatment group, and prevalence of comorbidity. Also shown the mortality of the reference group

	Total sample *n* = 1133		Men *n* = 736		Women *n* = 397		Age group 18–59 years *n* = 167		Age group 60–79 years *n* = 565		Age group ≥ 80 years *n* = 401		Operative *n* = 965		Non-operative *n* = 168		Non-operative *n* = 161*		No comorbidity *n* = 206		Reference group *n* = 4532	
Cumulative mortality	*n*	%	*n*	%	*n*	%	*n*	%	*n*	%	*n*	%	*n*	%	*n*	%	*n*	%	*n*	%	*n*	%
30 days	38	3.4	26	3.5	12	3.0	3	1.8	15	2.7	20	5.0	25	2.6	13	7.7	8	5.0	3	1.5	16	0.4
90 days	75	6.6	51	6.9	24	6.0	3	1.8	34	6.0	38	9.5	54	5.6	21	12.5	15	9.3	4	1.9	61	1.3
6 months	108	9.5	74	10.1	34	8.6	5	3.0	45	8.0	58	14.4	82	8.5	26	15.5	20	12.4	6	2.9	125	2.8
1 year	155	13.7	107	14.5	48	12.1	8	4.8	59	10.4	88	22.0	120	12.4	35	20.8	29	18.0	6	2.9	248	5.5
2 year	254	22.4	168	22.8	86	21.7	20	12.0	97	17.1	137	34.2	200	20.7	54	32.1	47	29.2	19	9.2	486	10.7

*The patients (*n* = 7) who were not offered surgery (because presenting in a moribund state) were not included

### Long-term excess mortality

**Fig. 1:**
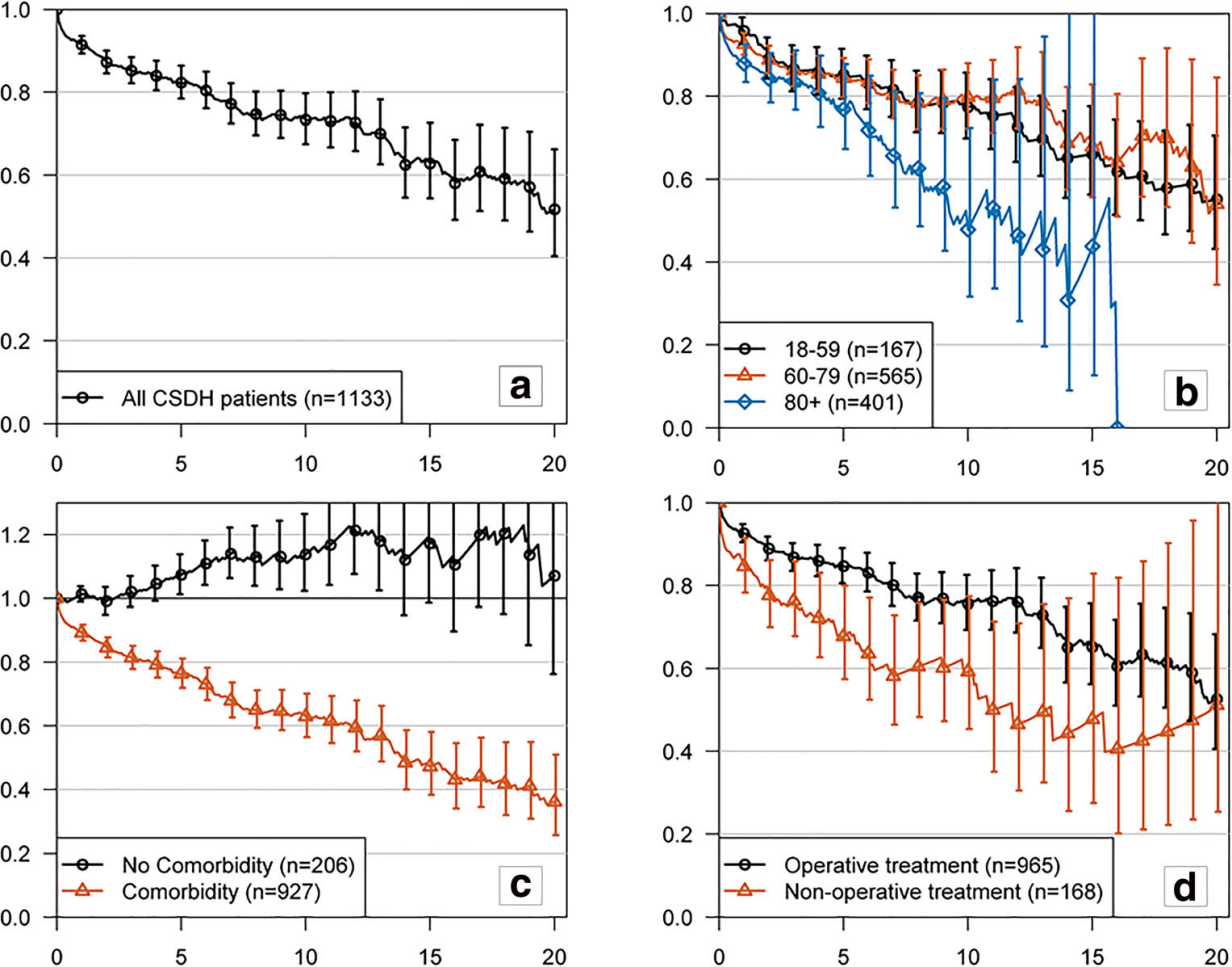
Excess mortality of chronic subdural hematoma patients. Cumulative relative survival ratios (with 95% confidence intervals) illustrating excess mortality of the CSDH patients compared with the matched general population; entire cohort (**a**), stratified by age groups (**b**), by prevalence of comorbidity (**c**), and by treatment group. The horizontal line at 1.0 represents the survival of the matched general population and curves below that line represent excess mortality of the study population. The vertical line shows follow-up time (years)

The 1-year cumulative relative survival ratio (CRSR) for all CSDH patients was 0.91 (95% CI 0.89–0.94), implying 9% excess mortality compared with the matched general population. The cumulative excess mortality was 18% in 5 years (CRSR 0.82; 95% CI 0.78–0.86), 27% in 10 years (CRSR 0.73; 95% CI 0.67–0.80), 37% in 15 years (CRSR 0.63; 95% CI 0.54–0.73), and 48% in 20 years (CRSR 0.52; 95% CI 0.40–0.66). The excess mortality rate was highest during the first year of follow-up after diagnosis of CSDH, and it was 2–4% per year during the rest of the follow-up period. The excess mortality seemed to be more pronounced in women, but the difference was not statistically significant. CSDH patients had excess mortality in every age group, and it was more pronounced in the age group of ≥ 80 years and in the non-operatively treated patients. A subgroup of patients with no comorbidities had better survival than the matched general population. The CRSR with 95% confidence intervals are shown in Fig. 1.

### Relative excess risk of death

In the age-, gender-, and follow-up time–adjusted regression model for RER, excess mortality was significantly related to (i) poor mRS 4–5 (RER = 4.93) at admission and especially (ii) at discharge (RER = 8.31), (iii) chronic alcohol abuse (RER = 4.47), (iv) warfarin medication (RER = 2.94), (v) age ≥ 80 years old (RER = 1.83), (vi) non-operative treatment (RER = 1.56; moribund patients *n* = 7 excluded), and (vii) non-traumatic etiology (RER = 1.69). Hematoma localization (unilateral/bilateral), thickness, or recurrence were not related to the excess mortality. Detailed RER results are presented in Table 4.

**Table 4:** Relative excess risk of death (RER) estimates and 95% confidence intervals for each subgroup of the 1133 chronic subdural hematoma patients adjusted for age, gender, and follow-up time

	Total sample n = 1133		RER	95% CI
	n	%		
Sex				
Men	736	65.0	1	Ref
Women	397	35.0	1.17	0.82–1.65
Age at CSDH diagnosis, years				
18–59	167	14.7	1	Ref
60–79	565	49.9	1.05	0.68–1.61
≥ 80	401	35.4	1.83	1.11–3.02
Chronic alcohol abuse				
No	1007	88.9	1	Ref
Yes	126	11.1	4.47	2.88–6.95
Traumatic etiology				
Yes	672	59.3	1	Ref
No	461	40.7	1.69	1.20–2.38
Antithrombotic medication				
None	655	57.8	1	Ref
Antiplatelet	268	23.7	1.20	0.72–2.01
Warfarin	187	16.5	2.94	1.91–4.54
Warfarin AND antiplatelet	23	2.0	3.24	1.35–7.75
Admission GCS				
13–15	1007	88.9	1	Ref
9–12	91	8.0	3.53	2.35–5.32
3–8	35	3.1	5.71	3.47–9.41
Admission mRS				
0–3	580	51.2	1	Ref
4–5	553	48.8	4.93	3.12–7.80
Hematoma localization				
Unilateral	876	77.3	1	Ref
Bilateral	257	22.7	0.74	0.46–1.18
Hematoma thickness, mm (missing *n* = 23)				
≤ 15 mm	376	33.2	1	Ref
16–25	474	41.8	0.69	0.48–1.01
> 25	260	22.9	0.59	0.34–1.01
Operative treatment				
Yes	965	85.2	1	Ref
No	168	14.8	1.77	1.18–2.65
No^1^	161	14.2	1.56	1.01–2.41
Discharge GCS^2^				
13–15	942	97.6	1	Ref
9–12	9	0.9	14.84	6.64–33.18
3–8	14	1.5	98.30	39.35–245.56
Discharge mRS^2^				
0–3	727	75.3	1	Ref
4–5	238	24.7	8.31	5.48–12.58
Recurrent hematoma^2^				
No	692	71.7	1	Ref
Yes	273	28.3	0.78	0.48–1.26

*RER* Relative excess risk of death, *CI* Confidence interval, *Ref* Reference, *GCS* Glasgow Coma Scale score, *mRS* Modified Rankin Scale score

^1^The patients (*n* = 7) who were not offered surgery because they presented in a moribund state were not included

^2^Includes only operatively treated patients

### Causes of death after CSDH

**Fig. 2:**
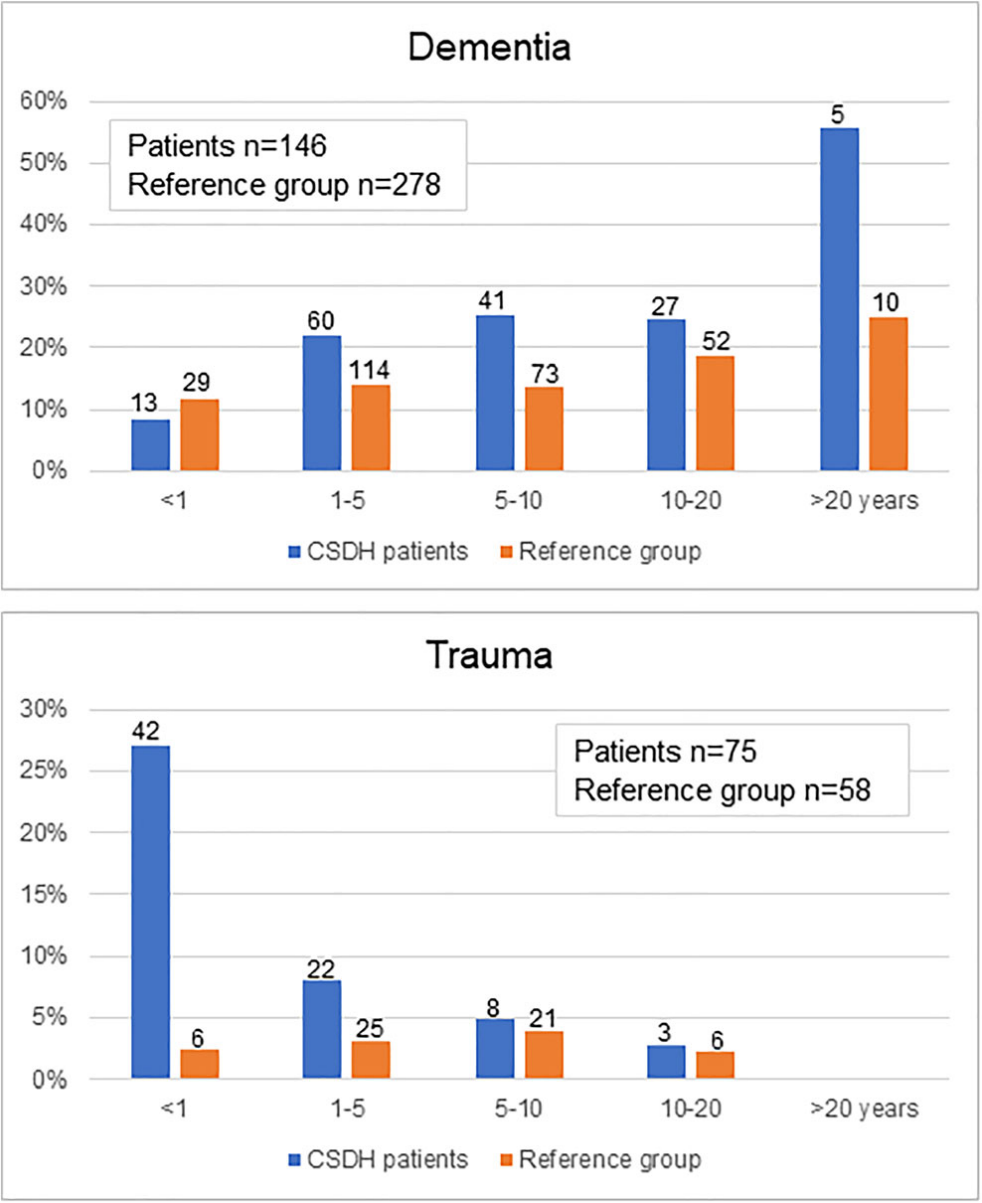
Dementia and trauma as a cause of death stratified by survival time. The CSDH patients are compared with the matched reference group. The percentages represent all the deaths during the time period. The number of deaths 710 among CSDH patients, and 1918 among the reference group

The most frequent causes of death for women were dementia (29%), ischemic cardiac disease (15%), and cerebral ischemia (12%), and the most common causes for men were ischemic cardiac disease (23%), dementia (16%), and cancer (14%). SDH (the hematoma type (chronic, subacute, acute) could not be verified due to the nature of the cause of death data) was the cause of death in 42/710 patients (6%). In the matched reference group (*n* = 4532), the most common causes of death were ischemic cardiac disease (25%; women 22% and men 26%), cancer (19%; women 15% and men 22%), and dementia (15%; women 18% and men 13%). As a cause of death, dementia was more common in patients with CSDH than in the reference group (21% vs. 15%, *p* < 0.001). The difference was significant for women (29% vs. 18%, *p* < 0.001), but not for men (16% vs. 13%, *p* = 0.12). The cause of death was traumatic in 11% of the CSDH patients, and 3% in the reference group (*p* < 0.001). SDH was the cause of death in 6% and in 0.5%, respectively (*p* < 0.001). Causes of death among the CSDH patients and the reference group are presented in Table 5. When stratifying the causes of death by survival time, the incidence of trauma-related death was significantly higher among CSDH patients compared with the reference group during the first 5 years (*p* < 0.001). In contrast, the incidence of dementia as the cause of death was higher among CSDH patients compared with the reference group after the first year, and increased over time reaching statistical difference from 1 to 10 years (*p* < 0.001; Fig. 2).

**Table 5:** The causes of death until the end of 2017 of the 1133 patients with chronic subdural hematomas between 1990 and 2015 in Pirkanmaa, Finland. The causes of death in the matched reference group between 1990 and 2015 are presented for comparison

	Total sample *n* = 1133		Men *n* = 736		Women *n* = 397		Operative treatment *n* = 965		Non-operative treatment *n* = 168		Causes of death in the matched reference group *n* = 4532	
	*n*	%	*n*	%	*n*	%	*n*	%	*n*	%	*n*	%
Age, years (median, IQR)	76	67–83	75	65–81	79	70–85	76	66–82	79	68–86	76	67–83
Follow-up time, years												
Median	4.8		5.1		4.6		5.2		3.3		6.4	
Range	0–27		0–26		0–27		0–27		0–21		0–28	
No. of deaths	710	62.7	449	61.0	261	65.7	609	62.3	101	60.1	1918	42.3
Age at death, median, IQR	84	76–89	83	74–88	86	80–91	84	76–89	85	79–90	84	78–88
Ischemic cardiac disease	143	20.1	103	22.9	40	15.3	122	20.0	21	20.8	473	24.7
Cerebrovascular disease	90	12.7	54	12.0	36	13.8	80	13.1	10	9.9	216	11.3
Cerebral hemorrhage	18	2.5	13	2.9	5	1.9	13	2.1	5	5.0	23	1.2
Cerebral ischemia	72	10.1	41	9.1	31	11.9	67	11.0	5	5.0	167	8.7
Cancer	91	12.8	64	14.3	27	10.3	80	13.1	11	10.9	370	19.3
Dementia and Alzheimer’s disease	146	20.6	70	15.6	76	29.1	121	19.9	25	24.8	278	14.5
Pulmonary disease	34	4.8	24	5.3	10	3.8	29	4.8	5	5.0	119	6.2
Pneumonia	16	2.3	9	2.0	7	2.7	15	2.5	1	1.0	45	2.3
Trauma	75	10.6	50	11.1	25	9.6	65	10.7	10	9.9	58	3.0
Accidental falls	41	5.8	25	5.6	16	6.1	36	5.9	5	5.0	33	1.7
Subdural hematoma, traumatic	38	5.4	25	5.6	13	5.0	33	5.4	5	5.0	9	0.5
Subdural hematoma, non-traumatic	4	0.6	4	0.9	0		3	0.5	1	1.0	1	0.05
Unknown	0		0		0		0		0		20	1.0

## Discussion

### Summary of the key findings

In our large population-based cohort, patients with CSDH had excess mortality, which increased over time from 9% at 1 year to 48% at 20 years after CSDH diagnosis. A subgroup of patients with no comorbidities had no excess mortality. The most important factors related to excess mortality were neurological disability at admission and at discharge. Age over 80 years almost doubled, warfarin almost tripled, and chronic alcohol abuse almost quintupled the risk of death after CSDH. Hematoma localization (unilateral/bilateral) or thickness were not relative risk factors nor was hematoma recurrence. In the median follow-up time of 4.8 years, there were 710 deaths, of which 6% were caused by SDH. The most common cause of death was dementia, which was significantly more common as a cause of death among the CSDH patients than in the reference group. As a cause of death, dementia occurred later in CSDH patients than in the reference group.

### Comparison of the current findings to prior literature

Miranda et al. observed excess mortality up to 1 year beyond diagnosis, but after that, life expectancy was equivalent with the general population [29]. Treatment group, size or laterality of subdural hematoma, and antithrombotic medication use were not associated with the mortality rate. Dumont and colleagues showed that patients with CSDH had worse survival than expected in every age group, and patients undergoing surgical drainage of CSDH (median survival 5.5 years) had significantly longer survival compared with patients not undergoing surgical drainage (2.3 years) [11]. The authors speculated that there can be selection bias, because the patients most likely to improve from surgery were offered surgical treatment. Mortality after CSDH was highest in the oldest patients over 85 years old, but the standardized mortality ratio was lower than in any other age group. Manickam et al. reported excess mortality continuing throughout a prolonged follow-up (median 5.2 years) as peers lived 12.4 years longer [25]. A prospective, randomized study by Santarius et al. revealed that among operatively treated patients, CSDH drainage significantly reduced 6-month mortality from 18 to 9% [33]. Additionally, a recent 5-year follow-up analysis of the aforementioned study showed a significant survival advantage for drainage as the relative survival in the no drain group was 77.6% compared with 89.8% in the drain group [16].

In our study, CSDH patients had excess mortality in every age group. The excess mortality was more pronounced in the age group of ≥ 80 years. The risk of excess mortality was higher in the non-operative group than in the operative group (RER 1.56) even though the neurological condition at admission was better among non-operatively treated patients, and they were not predisposed to surgical complications. The reason behind this is probably that the burden of comorbidities was somewhat higher among non-operatively treated patients.

In contrast to the study by Santarius, CSDH recurrence was not a risk factor for excess mortality among our study patients. In fact, the patients with recurrence had a lower mortality at least during the first 2 years. We speculate that this might be explained by more frequent medical attention (follow-ups and re-operations) for patients that are in better general health before their first CSDH. Patients with more comorbidities are less likely to undergo a second operation. Even so, our 6-month overall mortality rate (10%) was comparable with the findings by Santarius and colleagues [33]. Also, our relative survival at 5 years was similar (82%) than analyzed by Guilfoyle [16]. The mortality differences between studies are most likely due to differences in case ascertainment, healthcare systems, and population-related life expectancies.

Our study is in line with the previous studies demonstrating that neurological disability at discharge is strongly associated to long-term survival [11, 25, 29]. This is no surprise because it correlates to functional status, which has been recognized to have a great impact on life expectancy in general [20]. However, it is difficult to differentiate the effects of underlying comorbidities from the effects of CSDH on long-term survival. A subgroup of patients (*n* = 206) with no comorbidities survived better than the matched general population. In addition, hematoma bilaterality, thickness, or recurrence were not relative risk factors. Accordingly, even a large recurring CSDH may not affect the long-term survival by itself. Therefore, the patient-related variables are probably more important than the CSDH itself. Based on this data, it seems likely that the comorbidities are the cause of excess mortality rather than CSDH itself. Some patients are frail due to age-associated brain atrophy and other comorbidities, and CSDH seems to be a sentinel health event, a harbinger of subsequent morbidity and mortality, for this group of patients [3, 11, 29]. In contrast, patients with no comorbidities are probably healthier than the matched general population.

Hence, the excess mortality after diagnosis of CSDH might be reduced by more assertively treating the comorbidities, of which the most common were vascular diseases, diabetes, and chronic alcohol abuse. It is also known, that the hospitalization of older people decreases daily living functioning [7]. For this reason, it has been proposed that already perioperative care should be optimized by a multidisciplinary approach and by promoting early rehabilitation [35].

Antithrombotic drug use is common among CSDH patients and is speculated to attribute to the greater incidence of CSDH among elderly [6, 8, 15, 23, 30]. In our study, the use of warfarin, but not the use of antiplatelets, was a relative risk factor for excess mortality. This could reflect the increased risks of warfarin in the context of CSDH or be explained by the fact that the baseline diseases treated with antiplatelet drugs are not as severe as with warfarin. Similarly, chronic alcohol abuse was a relative risk factor for excess mortality after CSDH, but it is a risk factor for excess mortality also independently [9, 36]. Moreover, non-traumatic etiology was a relative risk factor for excess mortality among CSDH patients. This could be at least partly explained by the more common use of antithrombotic medication by patients with non-traumatic than traumatic etiology (47% vs. 39%), and the underlying medication-suggesting comorbidities. Additionally, CSDH might be a manifestation of degenerative or inflammatory disease rather than trauma [14]. In other words, aging, longstanding and ongoing alcohol abuse, and worse baseline general and neurological health all appear to contribute to greater mortality following CSDH.

The most important reason for the greater incidence of CSDH among the elderly has been speculated to be attributed to brain atrophy [22, 24, 37]. Dementia has been linked to brain atrophy [5]. At the time of diagnosis of CSDH, the prevalence of dementia in our CSDH patients (12% in patients aged 70 years or older) was similar as the prevalence in Western Europe reported by the World Alzheimer report 2015 [31]. As a cause of death, dementia was more prevalent in patients with CSDH than in our reference group. The difference was seen after the first year and was more pronounced in the later years. Additionally, the excess mortality related to CSDH increases with time. Our results support the idea that CSDH may be a risk factor for dementia. This could be explained by Bin Zahid and colleagues’ observation that CSDH is related to a significant increase in the degree of subsequent brain atrophy [4]. It seems that brain atrophy is a risk factor for CSDH, which in turn accelerates neurodegeneration and increases the risk of dementia. Further long-term prospective studies are needed to verify this association.

### Strengths and limitations

Our series represents the most extensive non-register-based study of consecutive CSDH cases treated in one neurosurgical department. Although retrospective in nature, the population-based setting makes it less prone to selection bias. Moreover, all the data was collected by one of the authors (M.R.). Our study gives a reliable population-based estimate of the CSDH-associated excess mortality based on the comparison with a matched general population.

This study has several limitations. ICD-codes were used to retrospectively identify all the patients of interest. There is a possibility that some cases were not recognized due to incomplete or incorrect ICD-coding. It is likely that all the patients undergoing surgery were identified, but the ICD-coding can be incomplete among the non-operatively treated patients, as a neurosurgeon has only been consulted on these cases. Neuroimaging was not reviewed, and there can be inconsistencies in reporting the hematoma thickness. In addition, the distinction between subacute and chronic SDH is not always obvious, both in relation to time and neuroradiological characteristics. No definition of CSDH is universally accepted [18]. Subacute SDHs were excluded from this study, because this hematoma subtype is considered to represent an entity of its own [2, 12, 21]. Additionally, autopsies were not performed on all of the deceased patients, and some of the causes of deaths might not be correct. Also, adjustments to the statistical analyses were limited because we did not have access to comorbidity data from the matched general population. However, it is reasonable to assume that controls and patients had similar comorbidities.

Future CSDH research should focus on preventive measures that take into account prior health conditions and fall-related injury risk factors that predispose to CSDH. Frailty, functionality, dependency, and comorbidity should be of special interest as these issues are prognosticators of general disability, hospital readmission, and mortality.

## Conclusions

Patients with CSDH have long-term excess mortality, which is evident up to at least 20 years after diagnosis. Patient-related characteristics, especially chronic alcohol abuse, antithrombotic medication use, and neurological disability both at admission, and at discharge, have a strong association with excess mortality, whereas specific CSDH-related findings do not. A subgroup of patients with no comorbidities had no excess mortality. The most common cause of death was dementia, which was more common as a cause of death in patients with CSDH than in the reference group after the first year. Consequently, there can be a two-way correlation between CSDH and dementia.
